# Comparison Between Prone SPECT-Based Semi-Quantitative Parameters and MBI-Based Semi-Quantitative Parameters in Patients with Locally Advanced Breast Cancer

**DOI:** 10.1007/s11307-024-01959-1

**Published:** 2024-11-08

**Authors:** Alina van de Burgt, Floris H. P. van Velden, Christinne L. S. Corion, Angela Collarino, Renato A Valdés Olmos, Frits Smit, Lioe-Fee de Geus-Oei, Lenka M. Pereira Arias-Bouda

**Affiliations:** 1 Department of Nuclear Medicine, Alrijne Hospital, Leiderdorp, The Netherlands; 2 Department of Radiology, Section of Nuclear Medicine, Leiden University Medical Center, PO Box 9600, 2300 RC Leiden, The Netherlands; 3 Department of Surgery, Haaglanden Medical Center, The Hague, The Netherlands; 4 Nuclear Medicine Unit, Fondazione Policlinico Universitario A. Gemelli IRCCS, Rome, Italy; 5 Biomedical Photonic Imaging Group, University of Twente, Enschede, The Netherlands; 6 Department of Radiation Science & Technology, Delft University of Technology, Delft, The Netherlands

**Keywords:** [^99m^Tc]Tc-sestamibi, Locally advanced breast cancer, Response prediction, Quantitative SPECT, SPECT/CT, Molecular breast imaging

## Abstract

**Purpose:**

This study evaluates the semi-quantitative single-photon emission computed tomography (SPECT) parameters of prone SPECT using [^99m^Tc]Tc-sestamibi and compares them with Molecular Breast Imaging (MBI)-derived semi-quantitative parameters for the potential use of response prediction in women with locally advanced breast cancer (LABC).

**Procedures:**

Patients with proven LABC with a tumor ≥ 2 cm on mammography and an indication for MBI using [^99m^Tc]Tc-sestamibi were prospectively enrolled. All patients underwent a prone SPECT/CT at 5 min (early exam) and an additional scan at 90 min (delayed exam) after injection of 600 MBq [^99m^Tc]Tc-sestamibi to compose wash-out rates (WOR). All patients underwent MBI after early SPECT/CT. Volumes of interest of the primary tumor were drawn semi-automatically on early and delayed SPECT images. Semi-quantitative analysis included maximum and mean standardized uptake values (SUV_max_, SUV_mean_,), functional tumor volume (FTV_SPECT_), total lesion mitochondrial uptake (TLMU), tumor-to-background ratios (TBR_max _and TBR_mean_), WOR and coefficient of variation (COV_SPECT_). Subsequently, the FTV_SPECT_, TBR_SPECT_ and COV_SPECT_ were compared to FTV_MBI_, TBR_MBI_ and COV_MBI_.

**Results:**

Eighteen patients were included. Early SUV_max,_ and TBR_max_ showed significantly higher interquartile range (IQR) compared to SUV_mean_ and TBR_mean_, respectively 2.22 (2.33) g/mL, 6.86 (8.69), 1.29 (1.39) g/mL and 3.99 (5.07) (median (IQR), *p* < 0.05). WOR showed a large IQR (62.28), indicating that there is WOR variation among the LABC patients. FTV showed no difference between MBI and early SPECT semi-quantitative parameter (*p* = 0.46).

**Conclusions:**

In LABC patients it is feasible to obtain semi-quantitative parameters from prone SPECT/CT. The FTV derived from early prone SPECT/CT is comparable with MBI-based FTV. Studies with comprehensive clinical parameters are needed to establish the clinical relevance of these semi-quantitative parameters, including WOR, for response prediction before its use in clinical routine.

**Supplementary Information:**

The online version contains supplementary material available at 10.1007/s11307-024-01959-1.

## Introduction

Molecular Breast Imaging (MBI), also previously referred to as breast specific gamma imaging (BSGI), provides a non-invasive *in vivo* characterization of breast lesions and is proven valuable for breast cancer detection, with sensitivity comparable to Magnetic Resonance Imaging (MRI) [1]. MBI holds a fundamental position when there is a contraindication for MRI or in situations when mammography and ultrasound have limited accuracy, such as in dense breasts, with free silicone (after silicone mastopathy) [2].

The radiopharmaceutical used for MBI is [^99m^Tc]Tc-methoxyisobutylisonitrile [^99m^Tc]Tc-sestamibi), which has been used in nuclear breast imaging for diagnosing breast cancer for over 20 years [3, 4]. [^99m^Tc]Tc-sestamibi has special characteristics, since it is a transport substrate for P-glycoprotein (Pgp) [5], encoded by the multidrug resistance gene that functions as energy-dependent efflux-pump for many drugs [6]. Therefore, reduced [^99m^Tc]Tc-sestamibi uptake in tumor cells might indicate Pgp over-expression, enabling upfront prediction of chemosensitivity. Determination of [^99m^Tc]Tc-sestamibi uptake during neoadjuvant chemotherapy (NAC) seems helpful in predicting non-responsiveness to NAC [7]. Therefore, quantification of [^99m^Tc]Tc-sestamibi accumulation might facilitate early *in-vivo* assessment of tumor chemoresistance and may guide treatment decision making.

Intra-tumor heterogeneity holds potential implications for tumor progression, treatment response, and therapeutic resistance [8]. Semi-quantitative [^99m^Tc]Tc-sestamibi parameters, as coefficient of variation (COV) and wash-out rates (WOR) [7, 9], are associated with this intra-tumoral heterogeneity [10, 11]. However, recent studies on semi-quantitative [^99m^Tc]Tc-sestamibi parameters revealed drawbacks in accurately assessing tumor uptake with planar MBI [12–14]. Single-photon emission computed tomography combined with computed tomography (SPECT/CT) might be helpful to overcome these drawbacks. It combines three-dimensional (3D) (whole-body) imaging with functional and anatomical information, compensating for tissue attenuation and scattering using low-dose CT and provides semi-quantification using [^99m^Tc]Tc-sestamibi [7, 15, 16]. To our knowledge, there are no clinical studies investigating the use of semi-quantitative SPECT parameters using [^99m^Tc]Tc-sestamibi in locally advanced breast cancer (LABC) patients. This prospective feasibility study aimed to evaluate the semi-quantitative parameters of prone SPECT/CT using [^99m^Tc]Tc-sestamibi and to compare them with MBI-derived semi-quantitative parameters for the potential use of response prediction in LABC patients.

## Methods

### Study Design and Patients

The Institutional Review Board approved this prospective monocenter study (trial code: NL60403.058.17). Between August 2017 and April 2019, all consecutive patients with pathologically proven LABC with a tumor ≥ 2 cm on mammography and ultrasound [16] and a clinical indication for local staging with MBI using [^99m^Tc]Tc-sestamibi [2] were included according to standard clinical procedures for pre-operative staging and to rule out multifocality. Although the SNMMI/EANM guideline [2] was published after our data collection, our study adhered to this. Patients who were pregnant or had undergone prior breast surgery, chemotherapy, or radiation therapy were excluded.

## Data Collection

### SPECT/CT Acquisition

Camera sensitivity was determined according to the vendor’s recommendations [15] as detailed in Collarino et al. [16]. Five minutes after injection with 600 MBq [^99m^Tc]Tc-sestamibi an early SPECT/CT was acquired. A second (delayed) SPECT/CT was acquired 90 min p.i. to compose the WOR [7]. The SPECT/CT scans were performed with a dual-head SPECT/CT gamma camera (Discovery NM/CT 670 Pro, GE Healthcare, Milwaukee, Wisconsin, USA). The patient was positioned in prone position (face down and arms up) using a supporting device for hanging breasts (hanging breasts mode) utilizing a single bed position. SPECT measurements were obtained with a low-energy, high-resolution (LEHR) collimator in noncircular orbit using step-and-shoot mode, over 360° (180° per head) and a 3° angular step with an acquisition time per frame/angle of 20 s (25 min in total). A 128 × 128 matrix size without zoom was applied and resulting in a voxel size of 4.42 × 4.42 × 4.42 mm^3^. The technetium energy window (photopeak) was set at 140.5 keV (window ± 10%) for emission and 120 keV (window ± 5%) for scatter. Consecutively, a low-dose CT was acquired for attenuation correction purposes with the patient breathing normally. The acquisition parameters include a tube voltage of 100 kV, a pitch of 1.375, a collimation of 20 mm and auto tube current modulation of 100 mA (30–150 mA).

All SPECT data underwent reconstruction using Evolution with Q.Metrix available on a Xeleris workstation version 4.DR (GE Healthcare, Milwaukee, USA) with an ordered subset expectation maximization (OSEM) algorithm that incorporates compensation for collimator-detector response, resolution recovery, attenuation, and scatter, using 9 iterations and 10 subsets [16]. The reconstructed voxel size of the SPECT images was 2.21 × 2.21 × 2.21 mm^3^. Additionally, CT data were reconstructed using an adaptive statistical iterative reconstruction (ASIR, GE Healthcare) algorithm with a voxel size of 2.21 × 2.21 × 2.21 mm^3^.

### MBI Acquisition

MBI was acquired directly after the early SPECT/CT scan according to standard procedure of our center [17]. Patients, while being seated, underwent five MBI (Dilon Diagnostics 6800, Pittsburgh, PA, USA) acquisitions (craniocaudal and mediolateral oblique of both breasts and lateral of the breast with tumor) of one frame with an acquisition time of 480 s (8 min) and a matrix size of 80 × 80, resulting in planer images with pixels of 3.20 × 3.20 mm.

## Image Analysis

### SPECT/CT Semi-Quantitative Parameters

SPECT/CT images were converted from counts to Bq/mL using Q.Metrix as previously detailed [16]. Volume of interest (VOI) of the primary tumors were semi-automatically delineated using a 42% threshold iso-contour method, followed by manual assessment by researcher (AB) to ascertain visual conformity [16]. Furthermore, approximately 3 cm diameter VOIs were manually drawn in the contralateral healthy breast and were used to estimate background activity. These VOIs were automatically projected to the co-registered SPECT images. Subsequently, the body-weighted mean, minimum and maximum standardized uptake values (SUV_mean_, SUV_max_; in g/mL), standard deviation (SD) of the mean SUV (in g/mL) and functional tumor volume (FTV; in mL) were measured. Furthermore, the total lesion mitochondrial uptake ($\text{TLMU}=\text{FTV}\times {\text{SUV}}_{\text{mean},\text{ tumor}}$), tumor-to-background ratio’s (${\text{TBR}}_{\text{max}}= \frac{{\text{SUV}}_{\text{max},\text{ tumor}}}{{\text{SUV}}_{\text{mean},\text{ background}}}$ and ${\text{TBR}}_{\text{mean}}= \frac{{\text{SUV}}_{\text{mean},\text{ tumor}}}{{\text{SUV}}_{\text{mean},\text{ background}}}$) and the wash-out rate ($\text{WOR}=\frac{{\text{TBR}}_{\text{early}}-{\text{TBR}}_{\text{late}}}{{\text{TBR}}_{\text{early}}}\times 100\text{\%}$) were composed [16, 18]. Next to these parameters, the coefficient of variation (${\text{COV}}_{\text{SPECT}}=\frac{{\text{SD SUV}}_{\text{mean},\text{tumour }}}{{\text{SUV}}_{\text{mean},\text{tumor }}}\times 100\text{\%}$) within the tumor was calculated to quantify a degree of tumor heterogeneity.

### MBI Semi-Quantitative Parameters

Quantification of [^99m^Tc]Tc-sestamibi uptake on MBI images was performed in Picture Archiving and Communication System (PACS; Sectra IDS7, Linköping, Sweden). Manual tumor delineations were performed on the MBI data by an experienced nuclear medicine physician (LP). An estimation of the FTV_MBI_ was acquired by performing manual tumor diameter delineations in three perpendicular axes (a,b,c) yielding: FTV_MBI_ = $\frac{4}{3}\times \pi \times a\times \frac{1}{2}\times b\times \frac{1}{2}\times c\times \frac{1}{2}$. The number of counts was obtained in three directions (cranial-caudal, mediolaterale-oblique and lateral) and the average was calculated. The counts in the background were determined by drawing a 3 cm diameter circle in the contralateral breast on the craniocaudal and mediolateral oblique projections.

The TBR on MBI was calculated in two ways. First, the TBR (TBR_max_) was calculated by dividing the maximum pixel value in the tumor by the highest mean pixel value of the background, in line with the SPECT TBR calculations. Secondly, the TBR (TBR_ave_) was calculated by dividing the average maximum pixel value of the tumor by the average pixel value of the background [9].

There are currently no standardized clinical protocols for calculating COV_MBI_. Therefore, COV_MBI_ was calculated in three ways in line with literature. The COV_max_ was calculated by dividing the highest SD in the tumor by the highest mean value in the tumor, multiplied by 100%. Secondly the COV_ave_ was calculated by dividing the average SD in the tumor (SD_ave_) by the average of the mean value in the tumor multiplied by 100%. Moreover, the COV_norm_ was calculated based on literature by (SD_ave_)/[(mean pixel value of tumor)/(average value of the background)] [10, 11].

## Statistical Analysis

A Shapiro–Wilk test was performed to evaluate the normality of the data. Non-paired data were analyzed with either an independent T test or Mann–Whitney U test, and continuous data were presented as mean (SD) or median (interquartile range), depending on normality. Paired data analysis used either the paired T-test or Wilcoxon Signed Rank test, also depending on normality. Box plots were used to visualize the distribution of the SPECT-based semi-quantitative parameters and the MBI-based semi-quantitative parameters. Scatter plots and the Spearman’s Rank correlation coefficient were used to explore their relation ranging from strong negative consistent relationship (-1), no consistent relationship (0) to strong positive consistent relationship (+ 1). Statistical analysis was conducted using GraphPad Prism (version 9.3.1; GraphPad Software, San Diego, California, USA) and Excel (version 2023; Microsoft, Redmond, USA).

## Results

### Patient Characteristics

This observational prospective study initially included 18 patients. Written informed consent was obtained from all participants. Patients’ characteristics are shown in Table 1.

**Table 1:** Patients’ characteristics

Variable	*N* = 18
Age (Y)	56.2 (10.3)
Tumor type	
NST	12
Lobular	6
Grade	
1	1
2	14
3	3
Size (mm)	24.8 (8.3)
Hormone receptor	
ER	16
PR	15
HER-2	0
TN	2

Age and size are presented in Mean (SD). *NST* no special type, *ER* estrogen receptor, *PR* progesterone receptor, *HER-2* human epidermal growth factor receptor 2, *TN* triple negative

### Semi-Quantitative Parameters

#### SPECT

**Fig. 1:**
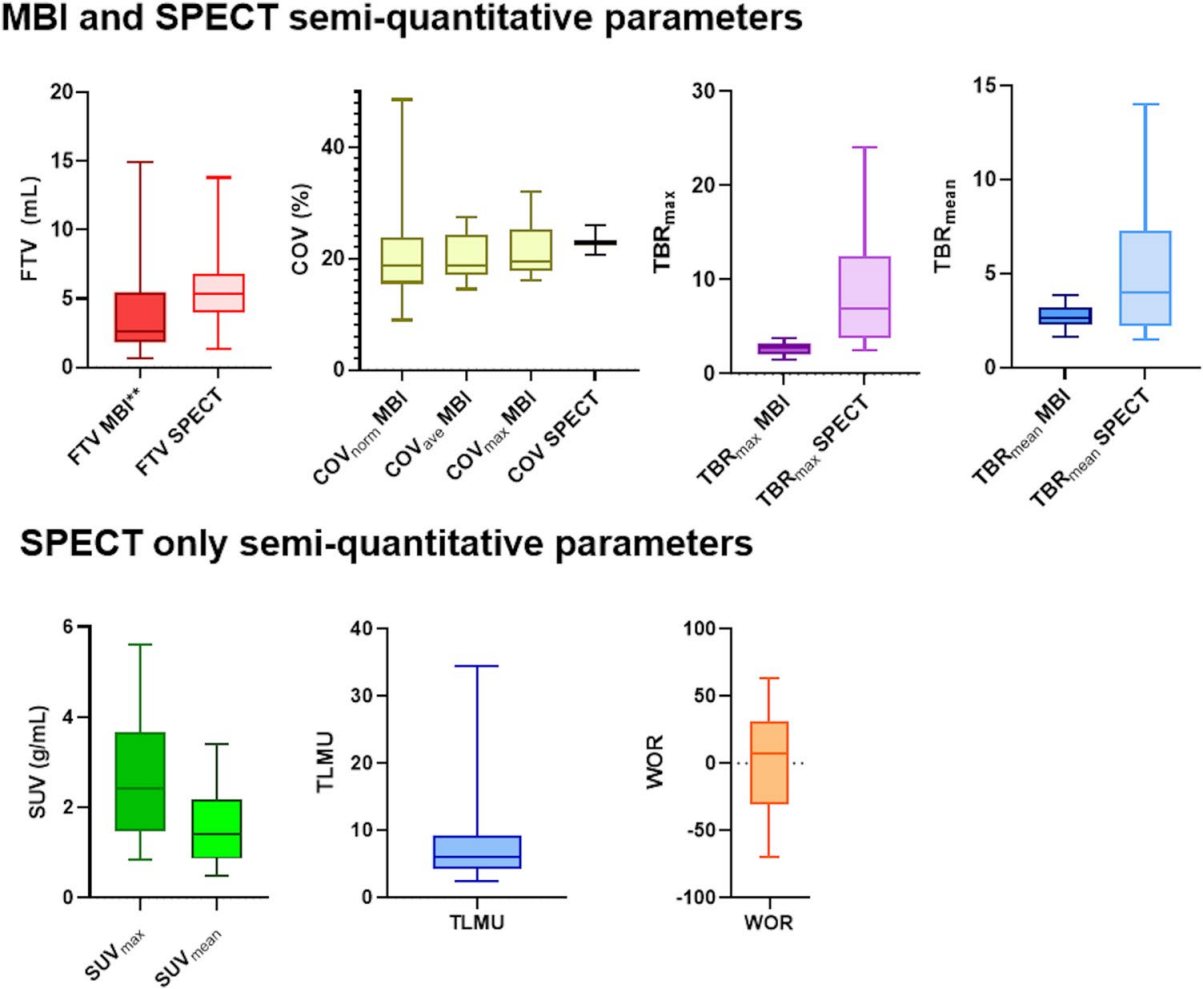
Semi-quantitative parameters SPECT and molecular breast imaging (MBI). The boxplots display the median (central line), 25th and 75th interquartile range (edges of the box), and the whiskers extending to the smallest and largest value for each semi-quantitative parameter. All patients underwent a prone SPECT/CT at 5 min (*N* = 18, early exam) and an additional scan at 90 min (*N* = 17*, delayed exam) after injection of 600 MBq [^99m^Tc]Tc-sestamibi to compose wash-out rates (WOR). MBI was acquired directly after the early SPECT/CT to directly compare the semi-quantitative parameters of both modalities. WOR varied significantly among patients as reflected by the large interquartile range. SPECT = single photon emission computed tomography; SUV = standardized uptake value; FTV = functional tumor volume; TLMU = total lesion mitochondrial uptake; TBR = tumor to background ratio. COV = coefficient of variation within the tumor. *The delayed acquisition was not performed for one patient due to technical difficulties. **FTV_*MBI*_* data of four patients were excluded because the tumor was not completely within the field-of-view (located close to the chest wall) or the tumor showed diffuse growth, making realistic volume dilations unfeasible.*

The semi-quantitative parameters of early and delayed SPECT acquisitions were calculated to assess the wash-out of [^99m^Tc]Tc-sestamibi (Fig. 1 and Supplementary Table S1). The delayed acquisition was not performed for one patient due to technical difficulties. Early SUV_max,_ and TBR_max_ showed significantly higher interquartile range (IQR) compared to SUV_mean_ and TBR_mean_, respectively 2.33(2.33) g/mL, 6.86(8.69), 1.29(1.39) g/mL and 3.99 (5.07) (median(IQR), *p* < 0.05). Note that WOR showed a large IQR (62.28), indicating that there is WOR variation among the LABC patients, see Fig. 1 and Supplementary Figure S1.

#### MBI

The MBI semi-quantitative parameters are presented in Fig. 1 and Supplementary Table S2. The FTV_MBI_ calculation was not possible for four patients because either the tumor was not completely within the field-of-view (located close to the chest wall) or the tumor had diffuse growth, making realistic volume dilations unfeasible. There was no significant difference between TBR_max_ and TBR_mean_ (*p* ≥ 0.05). The COV_ave_ showed the smallest IQR of 7.39% compared to COV_max_ and COV_norm_, respectively, 7.60% and 8.28% and was compared with COV_SPECT_ in the remainder of the study.

#### Comparison SPECT and MBI

**Fig. 2:**
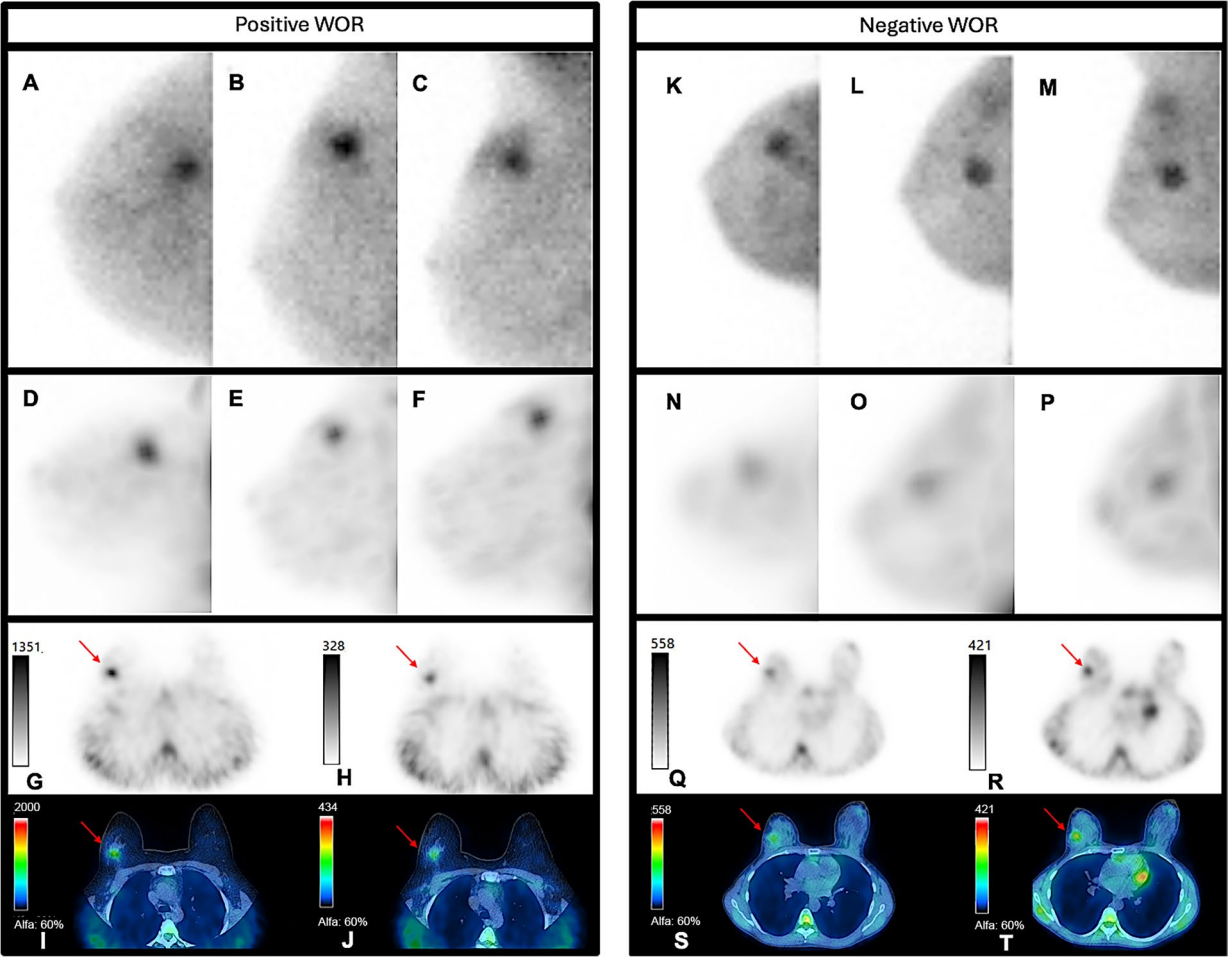
Molecular breast imaging (MBI) and single-photon emission computed tomography combined with computed tomography (SPECT/CT) images of two patients. The first patient (A to G) is a 45-year-old woman with invasive ductal carcinoma grade 3, estrogen receptor-negative, progesterone receptor-negative, and HER2-negative (triple-negative), MBI and SPECT/CT show high focal [^99m^Tc]Tc-sestamibi uptake of a 2 cm in diameter tumor located in the lateral upper quadrant of the right breast (red arrow) with visual lower uptake on delayed SPECT/CT and wash-out rate (WOR) of 18. The second patient (K to T) is a 43-year-old woman with invasive ductal carcinoma grade 3, estrogen receptor-positive, progesterone receptor-positive, and HER2-negative, MBI and SPECT/CT show moderate focal [^99m^Tc]Tc-sestamibi uptake of a 2 cm in diameter tumor located in the lateral upper quadrant of the right breast (red arrow) and visual increased uptake on the delayed images (WOR -33). MBI right craniocaudal view (A and K), MBI right lateral oblique view (B and L), MBI right mediolateral view (C and M), SPECT maximum intensity projection (MIP) right craniocaudal view (D and N), SPECT MIP right lateral oblique view (E and O), SPECT MIP right mediolateral view (F and P), axial SPECT of early (G and Q), and delayed (H and R) acquisition, fused axial SPECT/CT images of early (I and S) and delayed (J and T) acquisition.

Figure 2 shows comparable high focal [^99m^Tc]Tc-sestamibi tumor uptake in MBI and SPECT/CT images of two LABC patients. The two cases illustrate one patient with a positive WOR and one patient with a negative WOR, demonstrating a visual decrease and increase in uptake over time, respectively.

**Fig. 3:**
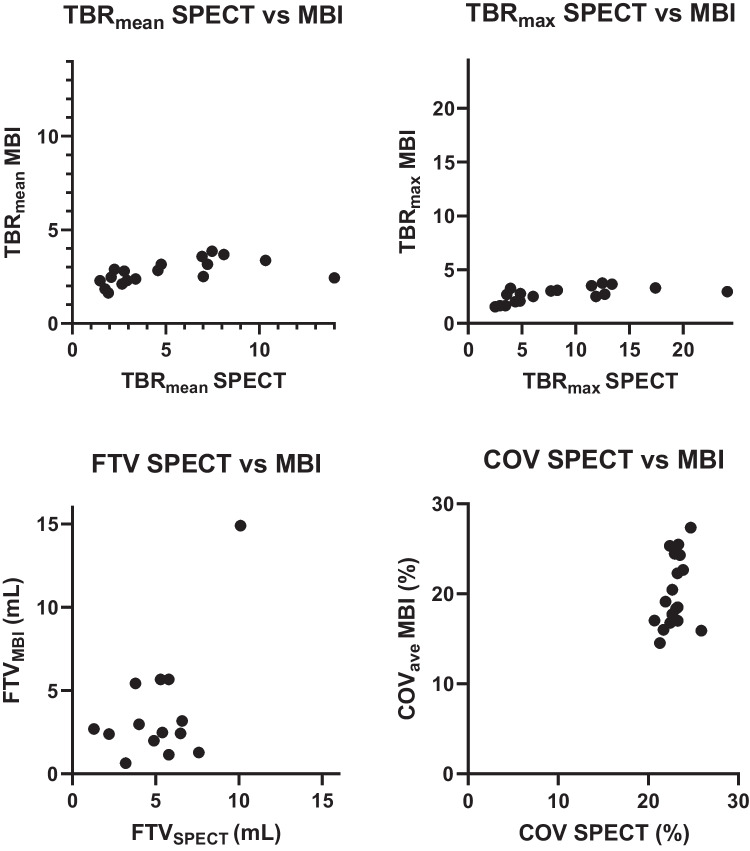
Scatter plots of SPECT versus MBI semi-quantitative parameters (*N* = 18). FTV = functional tumor volume; MBI = molecular breast imaging; SPECT = single photon emission computed tomography; COV = covariant of variation within the tumor; TBR = tumor to background ratio. Note that FTV_MBI_ data of four patients were excluded because the tumor was not completely within the field-of-view (located close to the chest wall) or the tumor showed diffuse growth, making realistic volume dilations unfeasible.

Various semi-quantitative parameters of the SPECT (early acquisition) and MBI are illustrated in Fig. 3. FTV_MBI_ data of four patients were excluded as explained previously. The TBR_max_ and TBR_mean_ revealed a significant difference (*p* < 0.05) between MBI and SPECT. TBR_mean_ showed a smaller median difference compared to TBR_max,_ respectively 1.34 and 4.12. The whiskers of the box plots for TBR_SPECT_ (mean and max) were significantly larger (*p* < 0.05) than those for TBR_MBI_, indicating greater variability of measurements for SPECT compared to MBI (Fig. 1). Spearsman’s correlation coefficient revealed a significant positive (*r* = 0.7, *p* < 0.05) consistent relation between MBI and SPECT, indicating that TBR_SPECT_ values were consistently higher compared to TBR_MBI_ (Fig. 3). FTV_SPECT_ did not show a significant (*p* = 0.46) difference compared to FTV_MBI._ The median difference between FTV_SPECT_ and FTV_MBI_ was 2.80 mL and the scatter plot indicated a diagonal trend, suggesting that the two methods provide comparable measurements. One patient showed a FTV higher than 10 mL compared to the others (approximately 5 mL). COV_SPECT_ were significantly larger (*p* < 0.05) compared to COV_MBI,ave_ and the variability in COV was significantly higher (*p* < 0.05) for MBI compared to SPECT (Figs. 1 and 3). However, visually, the values are comparable between the two techniques (Fig. 3). Spearman's rank correlation from FTV and COV between MBI and SPECT was not significant (*p* ≥ 0.05).

## Discussion

To our knowledge, this is the first feasibility study evaluating the semi-quantitative parameters of prone SPECT/CT using [^99m^Tc]Tc-sestamibi and comparing them with MBI-based semi-quantitative parameters in 18 patients with LABC. This study presents the first step towards a possible application of semi-quantitative parameters of prone SPECT/CT in LABC patients for prediction of response to NAC. Various semi-quantitative parameters were composed for early and delayed SPECT acquisitions (5 min p.i and 90 min p.i.) and MBI. No significant difference was observed between MBI and early SPECT semi-quantitative parameter FTV (*p* = 0.46). TBR_mean_ and TBR_max_ were significantly higher for SPECT compared to MBI and showed greater variability between the measurements (*p* < 0.05).

Early SUV and TBR_SPECT_ values were higher compared to late SUV and TBR_SPECT_, which probably is related to the clearance of [[^99m^Tc]Tc-sestamibi via transmembrane transporter proteins (like P-gp and the multidrug resistance protein (MRP)). In this regard, [^99m^Tc]Tc-sestamibi WOR is a promising predictive parameter for tumor non-responsiveness to NAC, as it reflects tumor multidrug resistance. Sciuto et al. reported high sensitivity and specificity for prediction of chemoresistance when applying a cut off WOR of 45% [19]. We were able to compose WOR derived from early and delayed SPECT/CT for potential future use in therapy response prediction.

The difference between FTV_SPECT_ and FTV_MBI_ might be explained by the FTV_MBI_ calculations assuming a spherical tumor, while in clinical practice, tumors exhibit various shapes. FTV_MBI_ data of four patients were excluded because the tumor was not completely within the field-of-view (located close to the chest wall) or the tumor showed diffuse growth, making realistic volume dilations unfeasible. These encountered limitations of MBI confirm the existence of challenges in achieving accurate tumor volume measurements when using MBI [12–14]. The increased variation between MBI and SPECT in tumors with higher average TBR_max_ and TBR_mean_ values might be attributed to the absence of attenuation and scatter correction in MBI compared to SPECT. Photon counts within tumor's VOI are affected by surrounding tissue (axilla e.g.), tumor specifications, breast properties, and imaging settings [14]. Consequently, the same tumor may appear differently across different views or detectors, resulting in variations in VOI measurements, which might affect the TBR calculations. Moreover, the lower TBR_MBI_ values are likely due to the higher septal penetration occurring by virtue of the 'near' contact imaging of the breast compared to the SPECT imaging. Therefore, the strengths of SPECT over MBI lie in its capacity for 3D imaging, especially for tumors located close to the chest wall and the clinical available SPECT attenuation and scatter correction, potentially composing more precise semi-quantitative parameters.

This study contains limitations. First, the limited number of subjects constitutes a major limitation and hence our results should be interpreted carefully. Although our study concerns only a small study population, we believe that our findings exhibit the complexity of assessing semi-quantitative SPECT/CT parameters and contribute to the knowledge of the application of [^99m^Tc]Tc-sestamibi for response prediction in LABC. A dynamic study should be conducted to evaluate which model best suits [^99m^Tc]Tc-sestamibi quantification and to relate the obtained pharmacokinetic measures to semi-quantitative measures obtained at different time intervals, hence examining their validity in this context. Second, the 42% threshold iso-contouring was utilized for the SPECT measurements based on a phantom study [16] since no protocols were available specifying the settings for quantitative SPECT with [^99m^Tc]Tc-sestamibi for LABC. Visual evaluation was conducted for the contouring, with manual adjustments if necessary, making delineations observer-dependent and thus affecting their reproducibility. Although Collarino et al. showed in a phantom study that absolute SPECT/CT quantification of breast studies using [^99m^Tc]Tc-sestamibi seems feasible (< 17% deviation) when 42% threshold iso-contouring is used for delineation of tumors (≥ 17 mm diameter) for various TBR_max_ (ranging from 9.6 to 3.3) [16], it is not clear how this 42% threshold iso-contouring would affect other semi-quantitative parameters in patients, such as FTV. Therefore, further investigation is necessary to determine which iso-contouring methods are most relevant and reproducible for clinically relevant semi-quantitative parameters in patients before applying quantitative SPECT for LABC in clinical settings. Third, outcome measures, such as pathologically confirmed therapy response, were not incorporated in this study. Before implementing response monitoring based on semi-quantitative SPECT with [^99m^Tc]Tc-sestamibi in clinical practice, the clinical relevance of SPECT-derived semi-quantitative parameters needs to be assessed in a future large prospective clinical trial, including histopathological response to NAC as primary outcome measure and gold standard. For this, the practice SPECT quantification guidelines [20], which were not available during our data collection but which overall principles align with our study, could be considered. Furthermore, before classifying a change in a semi-quantitative parameter as a response, it is crucial to assess its test–retest variability. Additionally, it is worthwhile investigating for which tumor molecular subtypes these parameters are more consistent.

## Conclusion

Obtaining semi-quantitative parameters of prone SPECT/CT using [^99m^Tc]Tc-sestamibi in women with LABC was feasible using 42% iso-contouring. No significant difference was observed between MBI and early SPECT semi-quantitative parameter FTV (*p* = 0.46). TBR_mean_ and TBR_max_ were significantly higher for SPECT compared to MBI and showed greater variability between the measurements (*p* < 0.05). Studies with comprehensive clinical outcome parameters are needed to establish the clinical relevance of these semi-quantitative parameters, including WOR, for response prediction, before it can be implemented in standard clinical care.

## Supplementary Information

Below is the link to the electronic supplementary material.

Supplementary file1 (DOCX 44 KB)

Supplementary file2 (DOCX 16 KB)

Supplementary file3 (DOCX 15 KB)

## Data Availability

The datasets generated during and/or analyzed during the current study are available from the corresponding author on reasonable request.
