# Supplementary material for: Comparison Between Prone SPECT-Based Semi-Quantitative Parameters and MBI-Based Semi-Quantitative Parameters in Patients with Locally Advanced Breast Cancer
Source: Mol Imaging Biol. 2024 Nov 8;26(6):926–33. doi: 10.1007/s11307-024-01959-1 (PMC11634910; doi:10.1007/s11307-024-01959-1)
Supplement: Supplementary file 1 — Supplementary file1 (DOCX 44 KB) [file 11307_2024_1959_MOESM1_ESM.docx]

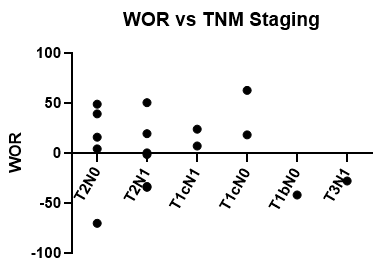


**Figure S1A. Scatter plot of wash-out rate (WOR) versus TNM (primary tumor [T], regional lymph nodes [N], distant metastases [M]) staging system** [1]. While pathological TNM (pTNM) is generally more accurate compared to clinical TNM (cTNM), it is unavailable for one patient due to surgery at a different hospital. For three patients who underwent neoadjuvant chemotherapy (NAC), cTNM is deemed more reliable. Accordingly, cTNM is used for patients who received NAC or had surgery at another institution, with pTNM applied to the remaining cases. The Spearman rank correlation coefficient between WOR and TNM is r=-0.07 but is not significant (p≥0.05). This suggests that there is no compelling evidence that the correlation is real and not due to chance.


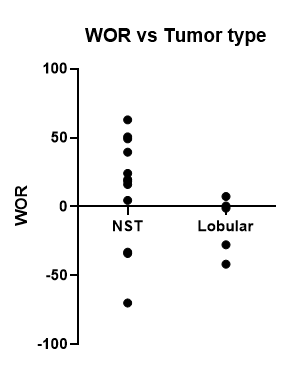


**Figure S1B. Scatter plot of wash-out rate (WOR) versus tumor type.** The Spearman rank correlation coefficient between WOR and Tumor type is r=-0.42 but is not significant (p≥0.05). This suggests that there is no compelling evidence that the correlation is real and not due to chance. NST= no special type.

1. Amin MB, Greene FL, Edge SB, et al. (2017) The eighth edition AJCC cancer staging manual: continuing to build a bridge from a population‐based to a more “personalized” approach to cancer staging. CA: a cancer journal for clinicians 67:93-99.
