# Supplementary material for: Comparison Between Prone SPECT-Based Semi-Quantitative Parameters and MBI-Based Semi-Quantitative Parameters in Patients with Locally Advanced Breast Cancer
Source: Mol Imaging Biol. 2024 Nov 8;26(6):926–33. doi: 10.1007/s11307-024-01959-1 (PMC11634910; doi:10.1007/s11307-024-01959-1)
Supplement: Supplementary file 2 — Supplementary file2 (DOCX 16 KB) [file 11307_2024_1959_MOESM2_ESM.docx]

**Table S1. Semi-quantitative parameters SPECT**

| Semi-quantitative parameters derived from the SPECT acquisitions | | | | |
| --- | --- | --- | --- | --- |
|  | SPECT Acquisition | |  |  |
|  | Early (N=18) | Delayed (N=17*) |  |  |
| SUV_max_ (g/mL) | 2.22 (2.33) | 1.65 (1.21) | ***p*=0.0044** | WOR 7.28 (62.28) |
| SUV_mean_ (g/mL) | 1.29 (1.39) | 0.93 (0.71) | ***p*=0.0013** |  |
| FTV (mL) | 5.40 (3.05) | 4.70 (3.55) | ***p***=0.9908 |  |
| TLMU | 5.89 (5.03) | 4.76 (4.32) | ***p*=0.0007** |  |
| TBR_max_ | 6.86 (8.69) | 5.59 (5.43) | ***p***=0.1743 |  |
| TBR_mean_ | 3.99 (5.07) | 3.37 (3.44) | ***p***=0.1883 |  |
| COV (%) | 23.08 (1.26) | 22.58 (1.79) | ***p***=0.7467 |  |

Values are presented as median (interquartile range) N=18. All patients underwent a prone SPECT/CT at 5 min (early exam) and an additional scan at 90 min (delayed exam) after injection of 600 MBq ^99m^Tc-sestamibi to compose wash-out rates (WOR). WOR varied significantly among patients as reflected by the large interquartile range. SPECT=single photon emission computed tomography; SUV=standardized uptake value; FTV=functional tumor volume; TLMU=total lesion mitochondrial uptake; TBR=tumor to background ratio. COV=coefficient of variation within the tumor; Early=acquisition 5 minutes after injection of ^99m^Tc-sestamibi; Delayed=acquisition 90 minutes after injection of ^99m^Tc-sestamibi. *p* <0.05 was deemed significant.

** The delayed acquisition was not performed for one patient due to technical difficulties*
