# Supplementary material for: Comparison Between Prone SPECT-Based Semi-Quantitative Parameters and MBI-Based Semi-Quantitative Parameters in Patients with Locally Advanced Breast Cancer
Source: Mol Imaging Biol. 2024 Nov 8;26(6):926–33. doi: 10.1007/s11307-024-01959-1 (PMC11634910; doi:10.1007/s11307-024-01959-1)
Supplement: Supplementary file 3 — Supplementary file3 (DOCX 15 KB) [file 11307_2024_1959_MOESM3_ESM.docx]

**Table S2. Semi-quantitative parameters molecular breast imaging (MBI)**

| Semi-quantitative parameters MBI | |
| --- | --- |
| FTV (mL)* | 2.60 (3.68) |
| TBR_max_ | 2.74 (1.21) |
| TBR_mean_ | 2.65 (0.93) |
| COV_max_ (%) | 19.51 (7.60) |
| COV_ave_ (%) | 18.82 (7.39) |
| COV_norm_ (%) | 18.83 (8.28) |

Values are presented as median (interquartile range) N=18. MBI=molecular breast imaging; FTV=functional tumor volume; TBR=tumor to background ratio; COV=coefficient of variation within the tumor.

**FTV_MBI_ data of four patients were excluded because the tumor was not completely within the field-of-view (located close to the chest wall) or the tumor showed diffuse growth, making realistic volume dilations unfeasible.*
